# Fractal dimension of chromatin is an independent prognostic factor for survival in melanoma

**DOI:** 10.1186/1471-2407-10-260

**Published:** 2010-06-05

**Authors:** Valcinir Bedin, Randall L Adam, Bianca CS de Sá, Gilles Landman, Konradin Metze

**Affiliations:** 1 Department of Pathology, Faculty of Medical Sciences, University of Campinas, Campinas, SP, Brazil; 2 Department of Pathology, A C Camargo Hospital, São Paulo-SP, Brazil

## Abstract

**Background:**

Prognostic factors in malignant melanoma are currently based on clinical data and morphologic examination. Other prognostic features, however, which are not yet used in daily practice, might add important information and thus improve prognosis, treatment, and survival. Therefore a search for new markers is desirable. Previous studies have demonstrated that fractal characteristics of nuclear chromatin are of prognostic importance in neoplasias. We have therefore investigated whether the fractal dimension of nuclear chromatin measured in routine histological preparations of malignant melanomas could be a prognostic factor for survival.

**Methods:**

We examined 71 primary superficial spreading cutaneous melanoma specimens (thickness ≥ 1 mm) from patients with a minimum follow up of 5 years. Nuclear area, form factor and fractal dimension of chromatin texture were obtained from digitalized images of hematoxylin-eosin stained tissue micro array sections. Clark's level, tumor thickness and mitotic rate were also determined.

**Results:**

The median follow-up was 104 months. Tumor thickness, Clark's level, mitotic rate, nuclear area and fractal dimension were significant risk factors in univariate Cox regressions. In the multivariate Cox regression, stratified for the presence or absence of metastases at diagnosis, only the Clark level and fractal dimension of the nuclear chromatin were included as independent prognostic factors in the final regression model.

**Conclusion:**

In general, a more aggressive behaviour is usually found in genetically unstable neoplasias with a higher number of genetic or epigenetic changes, which on the other hand, provoke a more complex chromatin rearrangement. The increased nuclear fractal dimension found in the more aggressive melanomas is the mathematical equivalent of a higher complexity of the chromatin architecture. So, there is strong evidence that the fractal dimension of the nuclear chromatin texture is a new and promising variable in prognostic models of malignant melanomas.

## Background

Malignant melanoma is a highly aggressive neoplasia of the skin with a constant and rapidly increasing incidence in the last decades [1] Prognostic factors are currently based on clinical data and morphologic examination (including variables such as tumor thickness, mitotic rate, etc.) [1-3], which are reliable and reproducible. Other prognostic markers, however, which are not yet used in daily practice, might add important information and thus improve prognosis, treatment, and survival. Therefore a search for new prognostic factors is desirable. For this purpose, traditional or new immunohistochemical markers, gene expression arrays, comparative genomic hybridization and mutational profiling have been applied [1,4-9]. Most of these techniques are sophisticated and expensive, requiring specially equipped laboratories with a trained staff, Detailed morphological analysis of the nuclei in histological or cytological preparations can give important information on cell physiology and, furthermore, can be of considerable great diagnostic and prognostic importance [10-22]. Physiologic or pathologic changes of the cell accompany changes of the chromatin arrangement [21,22]. In particular, neoplastic growth induces important modifications, not only of the DNA, but also of the composition and distribution of the histone and non-histone nuclear proteins, thus provoking alterations of the distribution of heterochromatin in the nucleus. Nuclear texture features have been studied as prognostic markers in neoplasias [11,21,22]. The resources available in commercial softwares are usually restricted to basic morphometric parameters such as diameter, area and perimeter, which cannot adequately measure texture features of nuclei.

An important aspect of texture analysis is the determination of fractality, since this feature characterizes the complexity of a structure not revealed by classical morphometry based on Euclidean geometry. Recent studies have shown the fractal nature of nuclear chromatin and of the surrounding nucleoplasmic space [23-25]. Fractals are self-similar structures, i.e. they exhibit similar features at different magnifications, in a scale-invariant manner. Previous studies have shown that fractal characteristics of nuclear chromatin are of prognostic importance in neoplasias [21,26-29]. Therefore we have investigated whether the fractal dimension of nuclear chromatin measured in routinely stained histological preparations of melanomas can be of prognostic survival value.

## Methods

### Study subjects

Patients who had superficial spreading cutaneous melanoma, diagnosed and treated at the Hospital AC Camargo, Center for Cancer Research and Treatment between 1994 and 2000 were examined. The study was approved by the local ethics committee (CONEP - 119149, 13th of December 2006.) Inclusion criteria were: 1. tumor size ≥ 1 mm; 2. paraffin blocks available for the construction of the tissue microarray; 3. detailed and complete clinical follow-up for at least 60 months in survivors. and 4. clinical information about the cause of death in non-survivors. All cases were reviewed according to the protocol established by the Brazilian Melanoma Group and the Brazilian Society of Pathology by a dermatopathologist (GL). The following histological variables were assessed: tumor thickness, Clark's level and mitotic rate (number of mitoses/mm^2^). Two core biopsies were obtained from paraffin-embedded tissue of each tumor within the previously identified and marked area. Using a Tissue Microarrayer (Beecher Instruments, Silver Spring, USA ™ ) with a sample needle of 1.0 mm, the tissue cores were transferred to a recipient paraffin block, according to the technique of Kononen et al [30]. A 6 μm section of the master block was stained with haematoxylin-eosin (H&E).

### Data collection

Image acquisition was performed with a Leica DC 500 ™ digital camera with high resolution (12 megapixels), and an oil immersion objective (x100). The images were saved without compression in bitmap format 24-bit color. At least 100 randomly selected tumor nuclei were acquired per case by the same operator. Only non overlapping nuclei with characteristics of melanoma cells were captured, interactively segmented and then converted to a 8 bit gray scale by calculating the luminance.

**Figure 1:**
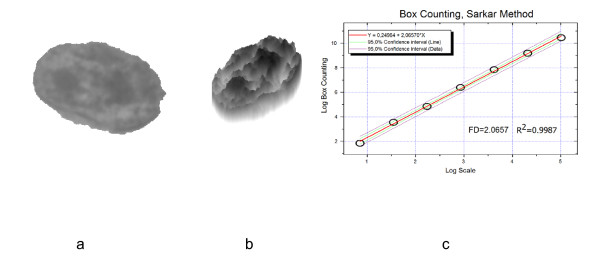
a) segmented nucleus b) pseudo-three-dimensional image, and c) log-log  diagram.

Finally we measured, as morphometric parameters of each nucleus, the nuclear area and the circular form factor. The latter represents the ratio between perimeter of a circle with the same area as the nucleus and the actual nuclear perimeter. The fractal dimension of the chromatin was calculated in accordance with Sarkar [31]. After normalization of the gray value histogram for each nucleus, a pseudo-three-dimensional image was created with the z axis defined by the gray level of each pixel, thus transforming the hematoxylin-stained chromatin texture into a rough surface (Fig1A, B). The fractal dimension (FD) of the surface of the normalized pseudo 3-D images was calculated with a software developed in house by our group. The space was filled with cubes and the number of intersections with the irregular landscape-like surface counted. This procedure was repeated with smaller or larger cubes. In a log-log (Fig 1C) diagram for each of these procedures, the number of intersections was plotted against the size of the cubes. The fractal dimension could be obtained by the slope of the linear regression line (Fig 1C). The goodness of fit of the regression line was estimated by the R^2 ^value of the regression between the real and the estimated values [32]. Furthermore, we tested with the Kolmogorov-Smirnov test, whether the residuals followed a normal distribution calculating for each patient the percentage of cells with normally distributed residuals.

### Statistical Analysis

Correlations between variables were calculated according to Pearson's method after testing for normal distribution with the Kolmogorov-Smirnov test. Melanoma specific survival was calculated with Cox regressions. Patient death not related to melanoma was censored. All variables with p ≤ 0.05 in the univariate Cox regressions were included in a multivariate proportional hazard model (p = 0.05 for input and p = = 0.1 for output, forward conditional step-wise selection) in order to get a prognostic model. Categorization of subgroups was not possible in the multivariate Cox model when there were no events in at least one of them, because, in this case, the regression did not converge. When this was the case, we defined each category by its quotient calculated between observed and expected events in the log-rank test of the corresponding Kaplan-Meier model, as suggested earlier [33].

## Results

The study involved 41 women and 30 men. The median follow-up was 104 months (8 -160). Fifteen patients died from disseminated disease. Primary tumors were located in the trunk in 47.1% patients, 44.1% in the extremities, and 8.8% in the head and neck. Tumor thickness, measuring 1.05 -17.0 mm (median 2.35 mm), was a significant adverse prognostic factor in the univariate Cox regression ( B = 0.1623; p = 0.0007). The Clark level ranged from 2 to 5, and was also a significant adverse prognostic factor ( B = 1.1574; p = 0.0011). At diagnosis, patients had a median age of 55 years (14-89). Age was not a prognostic factor in the univariate proportional hazard model (p > 0.10). Eight patients had lymph node metastases and 3 patients hematogenic metastases at diagnosis, but their survival was not statistically worse (p > 0.1 in both cases). The median mitotic rate was 1.875/mm^2 ^(0-32.5/mm^2^). The mitotic rate was significantly correlated with tumor thickness (r = 0.54; p < 0.0001) and revealed to be a negative prognostic factor (B = 0.0744. p = 0.007). The nuclear area (median value: 81.3 micra²; range 33.7 to 139.3) was also correlated both with the tumor thickness (r = 0.405; p = 0.00045) and the mitotic rate (r = 0.31; p = 0.008), and was a significant unfavorable prognostic factor (B = 0.0212; p = 0.0274).The form factor (median: 0.706 ranging from 0.57.3-0.807) was negatively correlated with tumor thickness (r = -0.431; p < 0.0001 ) and mitotic rate (r = -0.28; p = 0.016), but was of no prognostic relevance (p = 0.417). The fractal dimension of the chromatin structure ranged from 2.01 to 2.082 with a median of 2.06. The median of the R^2 ^values, which represent the goodness-of-fit of the regression lines, was 0.9998 (range 0.9989 - 0.9999). In each case, between 96% and 100% of the nuclei revealed normal distribution of the residuals. The fractal dimension was significantly correlated both with tumor thickness (r = 0.482; p < 0.0001) and the mitotic rate (r = 0.342; p = 0.002) and was a negative prognostic factor for survival (B = 73.9; p = 0.0091). We also ran a multivariate Cox regression, stratified for the presence or absence of metastases at diagnosis, and including all variables with p < 0.05 in the univariate models. The final regression included only Clark level (B = 1.0427; p = 0.0036) and fractal dimension of the chromatin (B = 55.169; p = 0.05) as independent prognostic factors.

## Discussion

The relevance of tumor thickness, Clark's level and the mitotic rate, which are well known prognostic factors in the literature [1,2,34], were confirmed in this study. But we could not demonstrate the presence of lymph node or hematogenic metastases as significant prognostic factors. This is certainly due to the very small number of patients with stage III or IV, so that the statistical test power was too low to show significant differences. Nevertheless, to eliminate even minor influences of metastases on the proportional hazard model, we stratified the Cox regression for the presence of metastases. The main emphasis of our investigation was to test karyometic variables being as prognostic factors.

The use of computerized image analysis has contributed to an objective description of melanoma cells and decreased substantially inter-observer variation. Objective measurement of nucleolar organizer regions (AgNORs), proliferating cell nuclear antigen (PCNA) and Ki-67, nuclear DNA content or karyometric variables such as chromatin compactness, and nuclear size and shape, have been used for differential diagnosis between melanomas and several forms of melanocytic nevi [35-37].

Karyometric differences between benign and malignant melanocytic lesions reflect alterations at the genetic and epigenetic level during the progression from common melanocytic nevi to dysplastic nevi to melanoma. This process involves dynamic changes in the genome produced by mutations through gain of oncogene function or loss of action of tumor suppressor genes. But many additional alterations of gene expression can be found during tumor progression, passing from the radial to the vertical growth phase and finally to the metastatic phenotype [1]. Abnormalities in mitotic regulators such as RASSF1A and Aurora kinases promote chromosomal instability on melanoma cells [38]. Comparative genomic hybridization revealed a higher number of genetic alterations in primary melanomas which developed metastases within one year after surgery compared to tumors that did not metastasize [39]. Thus the aggressive behavior of melanoma seems to be associated with an accumulation of multiple genetic alterations.

The nucleus is organized at three hierarchical levels: the organization of nuclear processes, the higher-order organization of the chromatin fiber, and the spatial arrangement of genomes within the nuclear space. Structure and function interact mutually in a dynamic way. There is increasing evidence for self-organization of nuclear architecture and function at all levels of organization leading to a high degree of structural complexity in the mammalian cell nucleus [40]. Chromatin is separated into two distinct conformations: euchromatin, which is uncondensed and the much denser heterochromatin, which is considered to be transcriptionally less active. Recent investigations are challenging this concept, demonstrating that chromatin structure is correlated with gene density, rather than activity. According to them, decondensed chromatin is representing gene-rich and condensed chromatin gene-poor regions [41]. Mascolo et al [42] demonstrated that overexperession of the chromatin assembly factor-1 which promotes histone incorporation into chromatin, was associatied with a higher risk to develop metastases. Based on these studies we may hypothesize that the chromatin organization of aggressive melanomas with an increased number of genetic and epigenetic alterations should be different from those with a more benign clinical course. The characteristics of the nuclear architecture, as seen by the pathologist in routine sections, reflect genomic and non-genomic changes in both the structure of DNA and chromatin [43]. Taking together all these studies, we may expect that changes of nuclear shape, size and chromatin texture would accompany tumor progression and the transformation to a more aggressive phenotype.

Our karyometric measurements corroborate this hypothesis. With increasing tumor thickness, i.e. vertical growth, the nuclear area enlarged and the form factor decreased, i.e. the nuclei tended to loose their roundedness. In the univariate Cox regression, nuclear area was also a significantly negative prognostic factor, i.e., the ability to metastasize was higher in melanomas with larger nuclei. This result confirms a previous study, which showed that the mean nuclear volume of primary melanomas with subsequent metastatic course was larger than tumors that did not metastatize [44]. Talve et al [45] observed in aneuploid melanomas a correlation of tumor thickness with nuclear size and increased genetic instability. The nuclear size in our investigation was also positively correlated with the fractal dimension of the chromatin architecture of the nuclei.

The fractal concept of self-similar structures was introduced by Mandelbrot [46]. Paumgartner et al. [47] applied it to microscopic images in biology and medicine. Grosberg et al [48] postulated that folded polymers should be fractals. During condensation, a polymer is repeatedly subjected to the self-similar process of crumpling, and this applies also to chromatin. Thus, the condensed polymer becomes finally a fractal. In that way a long polymer can be packed in a small volume without entanglements, which facilitates unravelling when necessary and is therefore an advantage for cell physiology [49]. Recent experimental studies showed indirect evidence for the fractal nature of DNA, nuclear chromatin and the surrounding nucleoplasmic space [23-25]. Our results gave evidence for the fractal nature of nuclear chromatin stained by haematoxylin in paraffin sections of melanoma cells.

The question whether a given structure should be considered as fractal, is linked not only to its scaling characteristics, which follow power laws. The goodness-of fit of the linear regression in the log-log plot is essential. The observed values should lie as close as possible on the straight regression line, where the slope gives an estimate of the fractal dimension. This condition was fulfilled since the R^2 ^values of the regression were allways > 0.99. Moreover, the histograms of the residuals, which are scattered around this line should follow a normal distribution [21,32,50]. In our study, between 96% and 100% of the nuclei per case had Gaussian distribution of the residuals. Therefore we are authorized to apply the concept of fractality and calculate the fractal dimension.

We are measuring the fractal dimension of a rough and irregular surface, The dimension of a plane surface is 2 and that of a cube 3. In an intuitive way one can interpret the fractal dimension of a rough, "landscape-like" surface as being in between the dimensions of a plane and of the cube, therefore 2 < FD < 3. The fractal dimension is equivalent to the slope of the regression line which depends on the number of intercepts of the surface with the boxes. A "smoother" surface has less intercepts and therefore a lower FD than a "rougher" surface. The fractal dimension was an independent adverse prognostic factor for survival in malignant melanomas. In other words, melanomas with a "rougher" surface had a bad prognosis. A more aggressive behaviour is usually found in genetically unstable neoplasias with a higher number of genetic or epigenetic changes, which provoke a more complex chromatin rearrangement, revealing an increased number of darker and lighter areas.

Previous studies demonstrated an association between higher FD values of the nucleus and worse prognosis in multiple myelomas [29], squamous cell carcinomas of the oral cavity [26] and in squamous cell carcinomas of the larynx [28].

Tumor thickness, nuclear size, mitotic index and fractal dimension have all been positively correlated with each other in this study. Nevertheless, the stepwise procedure in the multivariate Cox regression selected the fractal dimension and the Clark level as independent variables that built up the best proportional hazard model explaining patients' survival. This implies that the complexity of chromatin distribution contains relevant prognostic information which is independent of the Clark level. Since this investigation was based on a relatively small number of patients, it should be followed by confirmatory studies.

## Conclusion

Our study showed that the chromatin texture of hematoxylin-eosin stained nuclei in paraffin sections of melanoma cells can be described as a fractal. The increased nuclear fractal dimension found in the more aggressive melanomas is the mathematical equivalent of a greater complexity of their chromatin architecture. We conclude that there is strong evidence that the fractal dimension of the nuclear chromatin texture is a new and promising variable in prognostic models of malignant melanomas.

## Competing interests

The authors declare that they have no competing interests.

## Authors' contributions

VB participated in the study design, data collection and analysis, and participated in drafting and revising the manuscript. RLA elaborated the software, participated in the data analysis and critical revision of the manuscript. BCS participated in the data collection and analysis and critical revision of the manuscript. GL provided a histopathologic revision of the slides, participated in the data collection and the critical revision of the manuscript. KM conceived the study, was responsible for its design and coordination, participated in the analysis and interpretation of the data, as well as in drafting and revising all versions of the manuscript. All authors read and approved the final manuscript. The results presented in this publication are an essential part of the PhD thesis of VB, supervised by KM.(Postgraduate Course in Medical Pathophysiology, UNICAMP)

## Pre-publication history

The pre-publication history for this paper can be accessed here:

http://www.biomedcentral.com/1471-2407/10/260/prepub

## References

[B1] Carlson JA Ross JS Slominski AJ New techniques in dermatopathology that help to diagnose and prognosticate melanoma Clinics in Dermatology 2009 27 75 102 10.1016/j.clindermatol.2008.09.007 19095155

[B2] Gimotty PA Botbyl J Soong SJ Guerry D A population-based validation of the American Joint Committee on Cancer melanoma staging system J Clin Oncol 2005 23 8065 75 10.1200/JCO.2005.02.4976 16258105

[B3] de Sa BC Rezze GG Scramin AP Landman G Neves RI Cutaneous melanoma in childhood and adolescence: retrospective study of 32 patients Melanoma Res 2004 14 6 487 92 10.1097/00008390-200412000-00008 15577319

[B4] de Sá BC Fugimori ML Ribeiro Kde C Duprat Neto JP Neves RI Landman G Proteins involved in pRb and p53 pathways are differentially expressed in thin and thick superficial spreading melanomas Melanoma Res 2009 19 3 135 41 10.1097/CMR.0b013e32831993f3 19369901

[B5] Neto DS Pantaleão L de Sá BC Landman G Alpha-v-beta3 integrin expression in melanocytic nevi and cutaneous melanoma J Cutan Pathol 2007 34 11 851 6 10.1111/j.1600-0560.2007.00730.x 17944725

[B6] Bachmann IM Ladstein RG Straume O Naumov GN Akslen LA Tumor Necrosis is associated with increased alphavbeta3 integrin expression and poor prognosis in nodular cutaneous melanomas BMC Cancer 2008 8 362 10.1186/1471-2407-8-362 19061491 PMC2631589

[B7] Winklmeier A Poser I Hoek KS Bosserhoff AK Loss of full length CtBP1 expression enhances the invasive potential of human melanoma BMC Cancer 2009 9 52 10.1186/1471-2407-9-52 19216735 PMC2650708

[B8] Sousa JF Esperafico EM Suppression subtractive hybridization profiles of radial growth phase and metastatic melanoma cell lines reveal novel potential targets BMC Cancer 2008 8 19 10.1186/1471-2407-8-19 18211678 PMC2267200

[B9] Kaufmann S Kuphal S Schubert T Bosserhoff AK Functional implication of Netrin expression in malignant melanoma Cell Oncol 2009 31 6 415 22 19940358 10.3233/CLO-2009-0491 PMC4619052

[B10] Metze K Ferreira RC Adam RL Leite NJ Ward LS de Matos PS Chromatin texture is size dependent in follicular adenomas but not in hyperplastic nodules of the thyroid World J Surg 2008 32 12 2744 6 10.1007/s00268-008-9736-0 18787892

[B11] Ferreira RC de Matos PS Adam RL Leite NJ Metze K Application of the Minkowski fractal dimension for the differential diagnosis in thyroid Follicular neoplasias Cellular Oncology 2006 28 5 6 10.1155/2006/634840 PMC4617791 17167186

[B12] Mello MRB Metze K Adam RA Pereira FG Magalhães MG Machado Lorand-Metze I Phenotypic subtypes of acute lymphoblastic leukemia associated with different nuclear chromatin texture Analytical and Quantitative Cytology and Histology 2008 30 175 184 18561745

[B13] Moro-Rodríguez E Figols J Alvira M Uranga-Ocio JA García-Poblete E 13 GFAP and alpha1a-AR staining and nuclear morphometry of oligodendrogliomas by confocal microscopy and image analysis: useful parameters for predicting survival in oligodendrogliomas Diagn Pathol 2008 15 3 Suppl 1 S26 10.1186/1746-1596-3-S1-S26 PMC2500107 18673515

[B14] Adam RL Leite NJ Metze K Image preprocessing improves Fourier-based texture analysis of nuclear chromatin Anal Quant Cytol Histol 2008 30 3 175 84 18630843

[B15] Metze K Lorand-Metze IG Interpretation of the AgNOR pattern in hematologic cytology Acta Haematol 1993 89 2 110 2 10.1159/000204500 8503243

[B16] Li LX Crotty KA Palmer AA Kril JJ Stankovic R Scolyer RA McCarthy SW Differentiating benign nevi from malignant melanoma using DNA microdensitometry and karyometry and maturation: a zonal comparison, correlation and multivariate analysis Anal Quant Cytol Histol 2002 24 234 43 12199325

[B17] Li LX Crotty KA Palmer AA Kril JJ Scolyer RA Thompson JF McCarthy SW Argyrophilic staining of nucleolar organizer region count and morphometry in benign and malignant melanocytic lesions Am J Dermatopathol 2003 25 3 190 7 10.1097/00000372-200306000-00002 12775980

[B18] Cia EMM Trevisan M Metze K Argyrophilic nucleolar organizer region (AgNOR) technique: a helpful tool for differential diagnosis in urinary cytology 10.1046/j.1365-2303.1999.00145.x 10068885

[B19] Lorand-Metze I Carvalho MA Metze K Relationship between morphometric analysis of nucleolar organizer regions and cell proliferation in acute leukemias Cytometry 1998 32 51 56 10.1002/(SICI)1097-0320(19980501)32:1<51::AID-CYTO7>3.0.CO;2-I 9581624

[B20] Jondet M Agoli-Agbo R Dehennin L Automatic measurement of epithelium differentiation and classification of cervical intraneoplasia by computerized image analysis Diagn Pathol 2010 5 7 10.1186/1746-1596-5-7 20148100 PMC2819044

[B21] Adam RL Silva RC Pereira FG Leite NJ Lorand-Metze I Metze K The fractal dimension of nuclear chromatin as a prognostic factor in acute precursor B lymphoblastic leukemia Cell Oncol 2006 28 1-2 55 9 16675881 10.1155/2006/409593 PMC4615964

[B22] Montironi R Scarpelli M Lopez-Beltran A Mazzucchelli R Alberts D Ranger-Moore J Bartels HG Hamilton PW Einspahr J Bartels PH Chromatin phenotype karyometry can predict recurrence in papillary urothelial neoplasms of low malignant potential Cell Oncol 2007 29 1 47 58 17429141 10.1155/2007/356464 PMC4617991

[B23] Lebedev DV Filatov MV Kuklin AI Islamov AKh Kentzinger E Pantina R Toperverg BP Isaev-Ivanov VV Fractal nature of chromatin organization in interphase chicken erythrocyte nuclei: DNA structure exhibits biphasic fractal properties FEBS Lett 2005 579 6 1465 8 10.1016/j.febslet.2005.01.052 15733858

[B24] Lieberman-Aiden E van Berkum NL Williams L Imakaev M Ragoczy T Telling A Amit I Lajoie BR Sabo PJ Dorschner MO Sandstrom R Bernstein B Bender MA Groudine M Gnirke A Stamatoyannopoulos J Mirny LA Lander ES Dekker J Comprehensive mapping of long-range interactions reveals folding principles of the human genome Science 2009 326 289 293 10.1126/science.1181369 19815776 PMC2858594

[B25] Bancaud A Huet S Daigle N Mozziconacci J Beaudouin J Ellenberg J Molecular crowding affects diffusion and binding of nuclear proteins in heterochromatin and reveals the fractal organization of chromatin EMBO J 2009 28 3785 3798 10.1038/emboj.2009.340 19927119 PMC2797059

[B26] Goutzanis L Papadogeorgakis N Pavlopoulos PM Katti K Petsinis V Plochoras I Pantelidaki C Kavantzas N Patsouris E Alexandridis C Nuclear fractal dimension as a prognostic factor in oral squamous cell carcinoma. Oral Oncology 2008 44 4 345 353 17692559 10.1016/j.oraloncology.2007.04.005

[B27] Mashiah A Wolach O Sandbank J Lymphoma and leukemia cells possess fractal dimensions that correlate with their biological features Acta Haematologica 2008 119 142 150 10.1159/000125551 18417956

[B28] Delides A Panayiotides I Alegakis A Kyroudi A Banis C Pavlaki A Helidonis E Kittas C Fractal dimension as a prognostic factor for laryngeal carcinoma Anticancer Res 2005 25 3B 2141 4 16158956

[B29] Metze K Ferro DP Falconi MA Adam RL Ortega M Lima CP De Souza AC Lorand-Metze I Fractal characteristics of nuclear chromatin in routinely stained cytology are independent prognostic factors in patients with multiple myeloma Virchows Archiv 2009 455 S1 7 11

[B30] Kononen J Bubendorf L Kallioniemi A Bärlund M Schraml P Leighton S Torhorst J Mihatsch MJ Sauter G Kallioniemi OP Tissue microarrays for high- throughput molecular profiling of tumor specimens Nat Med 1998 4 844 7 10.1038/nm0798-844 9662379

[B31] Sarkar N Chaudhuri BB An efficient differential box-counting approach to computed fractal dimension of images IEEE Transactions on Systems Man and Cybernetics 1994 24 115 120 10.1109/21.259692

[B32] Metze K Lorand-Metze I Leite NJ Adam RL Goodness-of-fit of the fractal dimension as a prognostic factor Cell Oncol 2009 31 6 503 4 19940366 10.3233/CLO-2009-0505 PMC4619124

[B33] Lorand-Metze I Pinheiro MP Ribeiro E de Paula EV Metze K Factors influencing survival in myelodysplastic syndromes in a Brazilian population: comparison of FAB andWHO classifications Leukemia Research 2004 28 587 594 10.1016/j.leukres.2003.11.001 15120935

[B34] Homsi J Kashani-Sabet M Messina JL Daud A Cutaneous melanoma:prognostic factors. Cancer Control 2005 12 4 223 9 16258493 10.1177/107327480501200403

[B35] Li LXL Crotty KA Scolyer RA Thompson JF Kril JJ Palmer AA McCarthy SW Use of multiple cytometric markers improves discrimination between benign and malignant melanocytic lesions: a study of DNA microdensitometry, karyometry, argyrophilic staining of nucleolar organizer regions and MIB1- Ki67 immunoreactivity Melanoma Research 2003 13 581 586 10.1097/00008390-200312000-00007 14646621

[B36] Kossard S WilkinsoN B Nucleolar organizer regions and image-analysis nuclear morphometry of small-cell (nevoid) melanoma Journal of Cutaneous Pathology 22 2 132 136 10.1111/j.1600-0560.1995.tb01395.x 7560345

[B37] Rieger E Hofmann-Wellenhof R Soyer HP Kofler R Cerroni L Smolle J Kerl H Comparison of proliferative activity as assessed by proliferating cell nuclear antigen (PCNA) and Ki-67 monoclonal antibodies in melanocytic skin lesions. A quantitative immunohistochemical study J Cutan Pathol 1993 20 229 36 10.1111/j.1600-0560.1993.tb00648.x 8103531

[B38] Rother J Jones D Molecular markers of tumor progression in melanoma Curr Genomics 2009 10 4 231 9 10.2174/138920209788488526 19949544 PMC2709934

[B39] Balázs M Adám Z Treszl A Bégány A Hunyadi J Adány R Chromosomal imbalances in primary and metastatic melanomas revealed by comparative genomic hybridization Cytometry 2001 46 4 222 32 10.1002/cyto.1131 11514955

[B40] Misteli T Beyond the Sequence: Cellular Organization of Genome Function Cell 2007 128 4 787 800 10.1016/j.cell.2007.01.028 17320514

[B41] Gilbert N Boyle S Fiegler H Woodfine K Carter NP Bickmore WA Chromatin architecture of the human genome: gene-rich domains are enriched in open chromatin fibers Cell 2004 118 555 566 10.1016/j.cell.2004.08.011 15339661

[B42] Mascolo M Vecchione ML Ilardi G Scalvenzi M Molea G Di Benedetto M Nugnes L Siano M De Rosa G Staibano S Overexpression of Chromatin Assembly Factor-1/p60 helps to predict the prognosis of melanoma patients BMC Cancer 2010 10 1 63 10.1186/1471-2407-10-63 20178651 PMC2843674

[B43] Rothhammer T Bosserhoff AK Epigenetic events in malignant melanoma Pigment Cell Res 20 92 111 10.1111/j.1600-0749.2007.00367.x 17371436

[B44] Mossbacher U Knollmayer S Binder M Steiner A Wolff K Pehamberger H Increased nuclear volume in metastasizing "thick" melanomas J Invest Dermatol 1996 106 3 437 40 10.1111/1523-1747.ep12343580 8648173

[B45] Talve LA Collan YU Ekfors TO Primary malignant melanoma of the skin. Relationships of nuclear DNA content, nuclear morphometric variables, Clark level and tumor thickness Anal Quant Cytol Histol 1997 19 1 62 74 9051188

[B46] Mandelbrot BB Stochastic models for the Earth's relief, the shape and the fractal dimension of the coastlines, and the number-area rule for islands Proc Natl Acad Sci USA 1975 72 10 3825 3828 10.1073/pnas.72.10.3825 16578734 PMC433088

[B47] Paumgartner D Losa G Weibel ER Resolution effect on the stereological estimation of surface and volume and its interpretation in terms of fractal dimensions J Microsc 1981 21 Pt 1 51 63 10.1111/j.1365-2818.1981.tb01198.x 7230254

[B48] Grosberg AY Nechaev S Shakhnovich E The role of topological constraints in the kinetics of collapse of macromolecules J Phys France 49 2095 2100 10.1051/jphys:0198800490120209500

[B49] McNally JG Mazza D Fractal geometry in the nucleus EMBO Journal 2010 29 1 2 3 10.1038/emboj.2009.375 20051993 PMC2808382

[B50] Gonzato G Mulargia F Marzocchi W Practical application of fractal analysis: Problems and solutions Geophysical Journal International 1998 132 275

